# Circ-ABCA1 promotes oxidized low-density lipoprotein-induced inflammation and phenotypic switch in vascular smooth muscle cells

**DOI:** 10.1016/j.clinsp.2024.100343

**Published:** 2024-03-29

**Authors:** Fang Yu, JiWei Liu, Xiao Wei

**Affiliations:** a Department of Cardiac Catheterization Room, Yantaishan Hospital, Yantai City, Shandong Province, China; b Department of Emergency, Ezhou Central Hospital, Ezhou City, Hubei Province, China; c Department of 120 Emergency Center, The First People's Hospital of Jiangxia District, Wuhan City, Hubei Province, China

## Abstract

**Objective:**

Atherosclerosis (AS) is a chronic inflammatory disease of the arterial wall, in which Human Vascular Smooth Muscle Cells (HVSMCs) are involved. Nevertheless, the functions and mechanisms of circRNAs in oxidized Low-Density Lipoprotein (ox-LDL)-induced vascular smooth muscle cells remain unclear.

**Methods:**

Circ-ABCA1 expression was measured in the models of AS. Then, in the vitro model, oligonucleotide transfection was performed, followed by an analysis of VSMC proliferation, migration, inflammation, and phenotypic switch. Also, in the *in vivo* model, mice were injected with shRNA lentivirus, followed by histological examination of aortic tissues. Finally, the interaction of circ-ABCA1, miR-885–5p, and ROCK2 was identified.

**Results:**

Circ-ABCA1, was confirmed to be overexpressed in ox-LDL-induced VSMCs and mouse models of AS. Functionally, silencing circ-ABCA1 via oligonucleotide transfection suppressed VSMC proliferation, migration, inflammation, and phenotypic switch *in vitro* and prevented AS development in mice *in vivo*. Mechanistically, circ-ABCA1 absorbed miR-885–5p, which targeted ROCK2.

**Conclusion:**

Taken together, the data from this study suggest that circ-ABCA1 mediates cellular inflammation and phenotype switching through the miR-885–5p/ROCK2 axis in ox-LDL-induced VSMCs, and the circ-ABCA1/miR-885–5p/ROCK2 axis is a new potential biomarker for the treatment of AS.

## Highlights

• Si-circABCA1 inhibits VMSC malignant proliferation, inflammation, and phenotypic switch.
• miR-885–5p blocks the promoting effects of circABCA1 on VMSCs.
• CircABCA1 regulates the miR-885–5p/ROCK2 axis in VMSCs.
• Knockdown of circABCA1 reduces arterial plaque size and improves inflammation in AS mice.

## Introduction

Atherosclerosis (AS), originating from the intima, is a chronic inflammatory disease of the arterial wall and a major cause of cardiovascular disease, characterized by lipid metabolism disorders.^23^ Vascular Smooth Muscle Cells (VSMCs) have been believed to be key players in early and advanced AS.^8^ VSMCs are the main cells that make up the arterial media and are essential for maintaining the integrity of the arterial wall. Indeed, VSMCs participate in arterial wall remodeling, and cellular viability, migration, and phenotypic switch are of significance in the progression of AS.^13^^,^^6^ Therefore, exploring the action of VSMCs in AS is crucial for understanding the pathogenesis of AS and developing new therapeutic measures.

Circular RNA (circRNA) is a kind of endogenous non-coding RNA that is covalently closed and highly stable.^31^ Computational analysis of RNA sequencing has discovered thousands of circRNAs expressed in the cardiovascular system.^2^ Many circRNAs regulate gene expression by absorbing microRNA (miR) in the physiological and pathological processes of various diseases, including cardiovascular diseases.^15^ Circ-ABCA1 has been currently identified to be up-regulated after Spinal Cord Injury (SCI) and it exerts neurosuppressive effects when being silenced.^35^ However, whether circ-ABCA1 mediates AS has not been elucidated. miRs are capable of regulating a variety of physiological and molecular signaling pathways and mediating pathological and physiological changes in VSMC function.^24^ For example, miR-141–5p protects against AS in part by inhibiting VSMC inflammation, proliferation, and migration,^19^ and miR-33a-5p prevents VSMCs from ox-LDL-induced calcification.^10^ miR-885–5p has been confirmed to be abnormally expressed in elderly patients with cardiovascular disease and may prevent and treat cardiovascular disease.^25^^,^^29^ In addition, miR-885–5p has also been revealed to be involved in fat/lipid metabolism.^21^

The study targeted to explore the interrelationships of circ-ABCA1, miR-885–5p, and ROCK2 in AS by establishing AS cell models and animal models using human VSMCs and ApoE knockout mice. Ultimately, a novel molecular reference targeting AS may be developed based on the axis of circ-ABCA1/miR-885–5p/ROCK2.

## Materials and methods

### Clinical samples

Forty-six AS patients and 15 healthy subjects were recruited from Yantaishan Hospital between March 2017 and June 2019. All patients were diagnosed with carotid AS by angiography, and those with cancer and autoimmune or inflammatory diseases were excluded. The patient's blood samples were collected, and sera were obtained by centrifugation at 1000 × g and stored at −80 °C for RNA extraction. For human study, written informed consent and approval documents from the Ethics Committee of Yantaishan Hospital were obtained.

### Cell culture

Human VSMCs (ATCC, USA) were grown in Ham's F-12 K medium (Gibco/BRL life Technologies, Germany) containing 10 % fetal bovine serum (Hyclone, USA), 100 U/mL penicillin, and 100 mg/mL streptomycin, and placed under humidified conditions of 37 °C, 5% CO_2_. VSMCs were treated with 50 μg/mL Ox-LDL (Peking Union Biology Company, China) for 6 h, 12 h, and 24 h to establish an *in vitro* AS model.^14^

### Actinomycin D and RNase R treatment

The ring structure of circABCA1 was determined by actinomycin D and RNaseR treatment. Total RNA from VSMCs was incubated with 3 U/μg RNase R (Geneseed, China) or diethylpyrocarbonate-treated water (Sigma) at 37 °C.

To test for genetic stability, transfected VSMCs were treated with 4 μM actinomycin D for 0 h, 2 h, 4 h and 6 h, and analyzed by quantitative PCR to determine circABCA1 or GAPDH levels.

### Cell transfection

All transfection oligonucleotides/plasmids were obtained from GenePharma (Shanghai, China), including small interfering RNAs targeting circABCA1 and ROCK1 (si-circABCA1: ATGAATGGAGGAGGGAGAGC, si-ROCK2: GTCCTTGATTTAGATTTTCCTGC), overexpression vectors (oe-circABCA1, oe-ROCK2), miR-885–5p mimic/inhibitor, and the corresponding negative controls. Oligonucleotides (30 nM) or plasmids (100 ng) were transfected into VMSCs using Lipofectamine 2000 (Invitrogen). Quantitative PCR or immunoblotting was required to examine the transfection efficacy at 48 h post-transfection.

### CCK-8 analysis

VMSCs were allowed to grow in 96-well plates containing DMEM (100 μL/well) at 2 × 10^3^ cells/well for 0 h, 24 h, 48 h, and 72 h. At each time point, 10 μL of CCK-8 solution (Dojindo, Japan) was co-cultured for 2 h avoiding light at 37 °C. Absorbance was measured by a microplate reader (Bio-Rad, Hercules, CA) at 450 nm.

### ELISA

To evaluate the inflammatory response of VMSC, VMSCs were lysed and the supernatants were collected to measure IL-1β, IL-6, and TNF-α using commercial assay kits (Thermo Fisher Scientific, USA).

### Wound healing assay

Wound-healing assay was used to assess VSMC migration. VSMCs were seeded to generate confluent monolayers in 6-well plates and wounds were created by scraping cells with a 200 μL sterile pipette and were conditioned to recovery for 48 h. At that time, images of wounds were captured with a microscope.

### RT-qPCR

Total RNA from sera, mouse aortic tissue, and VMSCs was extracted by TRIzol reagent and reverse transcribed using the PrimeScript RT Master Mix kit (Takara). In the PCR system, SYBR® Green PCR Master Mix (Vazyme Biotech) was utilized, and the reaction volume was 10 µL using 5 µL 2 × PCR master mix (SYBR Premix Ex Taq), 0.5 µL PCR Primer, and 2 µL cDNA diluted to 20 µL ddH_2_O. Gene expression was calculated using the 2^−ΔΔCt^ method and normalized to GAPDH. Primers are shown in Table 1.

**Table 1:** PCR primer sequence.

	Primer sequence (5′‒3′)
**hsa_circ_0137614**	Forward: 5′- TGAAGCCAATCCTGTTAATGACC-3′
	Reserse: 5′-GGGTAACGGAAACAGGGGTT-3′
**mmu_circ_0011675**	Forward: 5′- CCAGTTGTGTTAATGACCAGCC-3′
	Reserse: 5′- TTCGTAGGGTGGGTAGCTCA-3′
**miR-885–5p**	Forward: 5′- TCCATTACACTACCCTGCCTCT −3′
	Reserse: 5′- TGGTGTCGTGGAGTCG-3′
**ROCK2**	Forward: 5′-GGCTTGTATGAAGCCCCTGT-3′
	Reserse: 5′-ACCCACTTCTGCTGCTCTTC-3′
**Human GAPDH**	Forward: 5′- CACCCACTCCTCCACCTTTG-3′
	Reserse: 5′- CCACCACCCTGTTGCTGTAG-3′
**mouse GAPDH**	Forward: 5′- CATCAACGGGAAGCCCATC-3′
	Reserse: 5′- CTCGTGGTTCACACCCATC-3′
**U6**	Forward: 5′-CTCGCTTCGGCAGCACA-3′
	Reserse: 5′-AACGCTTCACGAATTTGCGT-3′

### Western blot

Mouse aortic tissue and VMSCs were lysed with RIPA lysis buffer (Beyotime, China), and the products were analyzed by a BCA kit (Cowbiotech, China). After gel-separation by SDS-PAGE, 30 μg of protein was loaded onto a polyvinylidene fluoride membrane (Millipore, USA), followed by addition of 5 % bovine serum albumin and incubation with primary antibodies overnight at 4 °C including p65 (sc-8008, Santa Cruz Biotechnology), p-p65 (3033, Cell Signaling Technology), Ki-67 (ab15580, Abcam), α-SMA (A5228, MilliporeSigma), OPN (ab8448, Abcam). Afterward, Horseradish Peroxidase (HRP)-conjugated secondary antibody (ab6721; Abcam) was added to develop bands which were then scanned using Image Lab software (Bio-Rad).

### Dual-luciferase reporter assay

Fragments of Wild-Type (WT) or Mutant (MUT) circABCA1 and ROCK2 3′UTR containing the miR-885–5p binding site were synthesized and cloned into pmirGLO vector (Promega, USA) to generate recombinant luciferase vector (WT/MUT-circACBA1, WT-MUT-ROCK2). After co-transfection with miR-885–5p mimic or mimic NC in VSMCs according to Lipofectamine 2000, the luciferase activity of the recombinant luciferase reporter was determined using a dual-luciferase reporter assay system (Promega). The relative firefly luciferase activity was calculated by normalizing to renilla luciferase activity.

### RIP experiment

RIP analysis was performed by EZ-Magna RIP kit (Millipore, USA). VSMCs were made into lysates using RIP lysis buffer and combined with protein A/G magnetic beads and antibodies against IgG (Millipore) or AGO2 (Abcam). Then, purified RNA was obtained from a bead binding complex for quantitative PCR to measure RNA abundance.

### Animal experiment

Animals underwent treatment with the approval of the Animal Ethics Committee of Yantaishan Hospital. Twenty male ApoE^−/-^ mice (5‒6 weeks old, SJA Animal Experiment Co., Ltd., China) were randomized into 4 groups (*n* = 5): 1 normal diet group (10 % fat, 20 % protein, 70 % carbohydrate) and 3 high-fat diet groups (60 % fat, 20 % protein, 20 % carbohydrate). All mice were fed for 12-weeks at 20‒25 °C with a 12/12 light-dark cycle in the SPF environment. shRNA lentivirus targeting circABCA1 (sh-circABCA1, GenePharma) or the negative control were injected via tail vein (1 × 10^9^ PFU) into mice fed a high-fat diet every 3-weeks. Atorvastatin (AVT, 10 mg/kg) was administrated daily as a positive control. Finally, the mice were exposed to excess CO_2_, and the mouse aortic tissues were collected, some were fixed with 4% paraformaldehyde, and the rest were placed at −80 °C.

### HE staining

Aortic pathology was assessed as previously described.^11^ The tissue was embedded in paraffin, cut into 5 μm sections, and stained with HE staining solution.

### IHC staining

Aortic tissues were dewaxed in xylene, blocked with 3% H_2_O_2_, and added with 5% BSA for 15 min. Primary antibodies α-SMA (A5228, MilliporeSigma) and OPN (ab8448, Abcam) were supplemented to the tissues at 4 °C overnight, followed by HRP-labeled IgG secondary antibody for 1 h. Finally, DAB development was implemented for 10 min.^33^

### Data analysis

Statistical analysis was performed using GraphPad Prism 9.0 and data collected from at least three independent experiments were shown as mean ± Standard Deviation (SD). Two sets of data were compared by Student's *t*-test, and multiple sets were by one-way ANOVA; *p <* 0.05 was considered a significant difference.

## Results

### CircABCA1 expression at a high level in AS

**Fig. 1:**
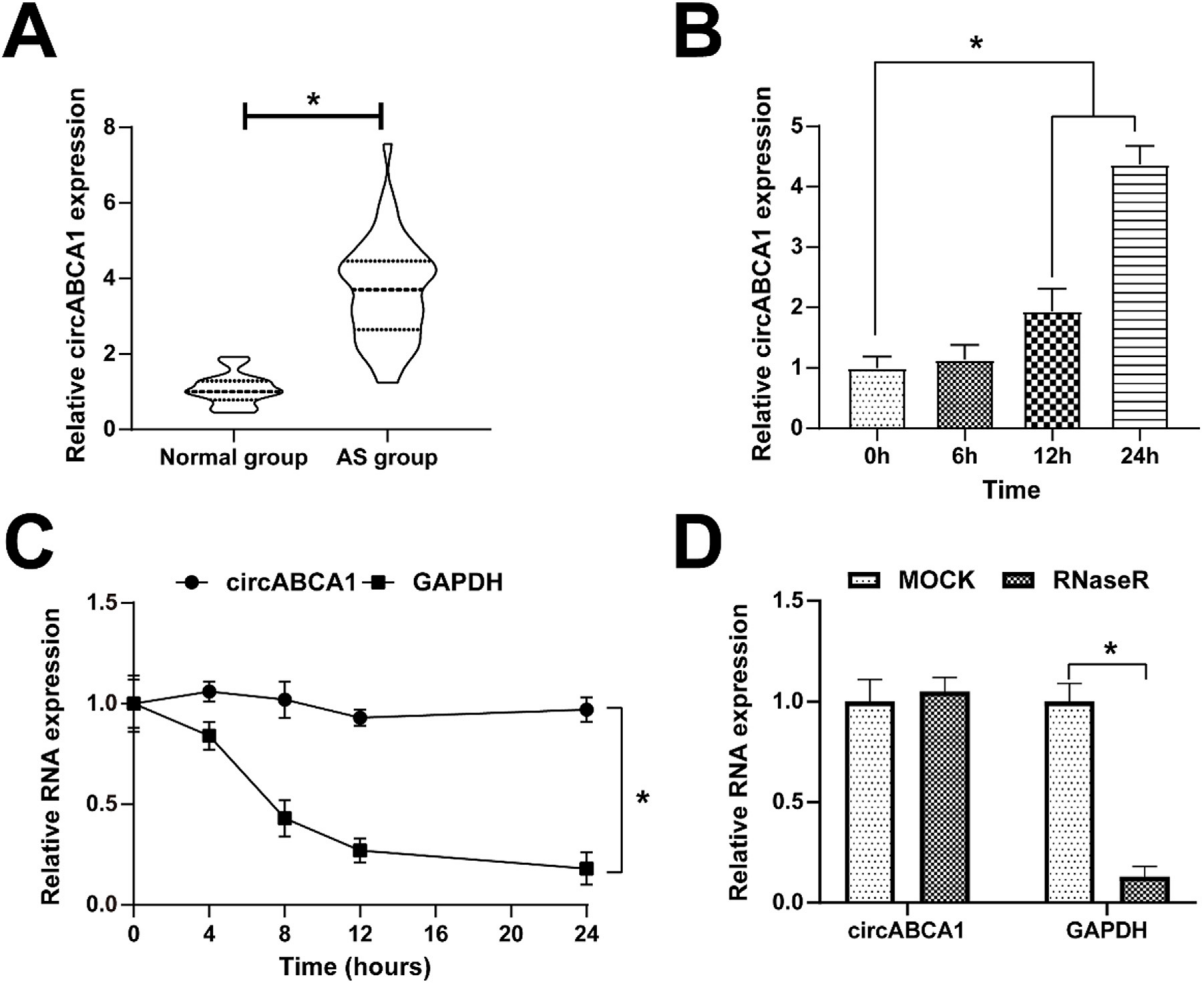
**CircABCA1 expression maintains a high level in AS.** CircABCA1 expression in patients’ sera (A) and VMSCs treated with ox-LDL for 0 h, 6 h, 12 h, and 24 h (B); The stability of circABCA1 (C) and the ring structure of circABCA1 (D); data are expressed as mean ± SD (*n* = 3); *p < 0.05.

Firstly, the expression of circABCA1 in AS was examined. CircABCA1 levels were higher in sera of AS patients than in healthy subjects (Fig. 1A). Since the formation of atherosclerotic plaques has an important relationship with the malignant proliferation of VMSCs, abnormally expressed circABCA1 is considered to relate to the malignant proliferation of VMSCs. Therefore, ox-LDL was used to induce the proliferation of VMSC, and the changes of circABCA1 were examined by quantitative PCR. Fig. 1B demonstrates the increase in circABCA1 levels after 12 h and 24 h of ox-LDL treatment. The ring structure of circABCA1 was examined by actinomycin D and RNase R experiments. As the results suggested, circABCA1 had a longer half-life than linear GAPDH mRNA, and RNsae R degraded linear GAPDH mRNA, but had no effect on circABCA1 expression (Fig. 1C‒D). In summary, circABCA1 has a ring structure and is highly expressed in AS.

### Si-circABCA1 inhibits vmsc malignant proliferation, inflammation, and phenotypic switch

**Fig. 2:**
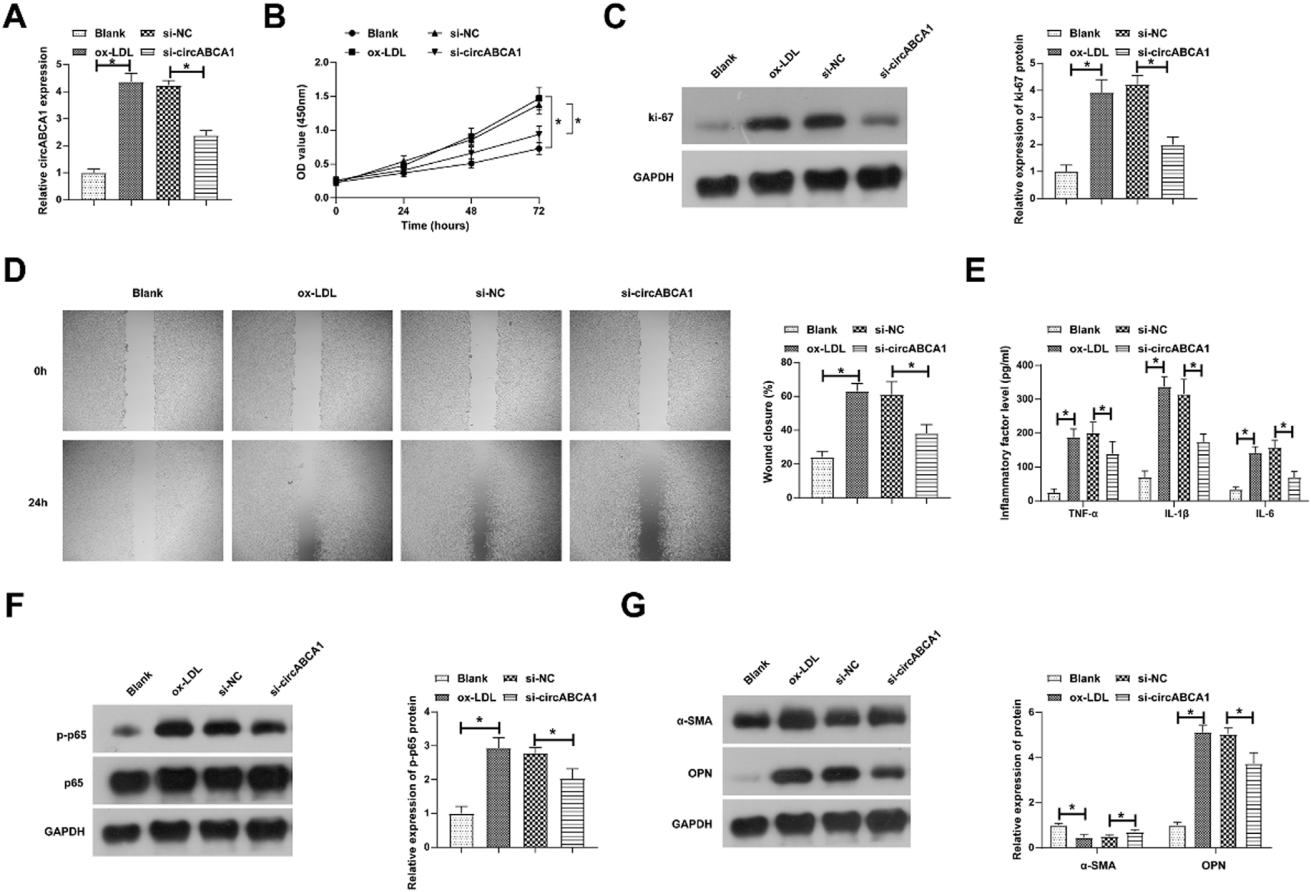
**Si-circABCA1 inhibits VMSC malignant proliferation, inflammation, and phenotypic switch.** si-circABCA1 was transfected into ox-LDL-treated VMSCs. Changes in circABCA1 expression (A), proliferation (B), Ki-67 protein expression (C), migration ability (D), levels of inflammatory factors (E), p-NF-κB protein expression (F), α-SMA and OPN protein expression (G); data are expressed as mean ± SD (*n* = 3); *p < 0.05.

Subsequently, circABCA1-targeting siRNA was transfected into ox-LDL-treated VMSC to investigate the biological function of circABCA1. Transfection of si-circABCA1 reduced circABCA1 levels in VMSCs (Fig. 2A). CCK-8 assay showed that ox-LDL treatment significantly promoted the proliferation of VMSC, but circABCA1 knockdown inhibited the proliferation of VMSC (Fig. 2B). In addition, the promoting effect of ox-LDL on the proliferation protein Ki-67 was alleviated by knockdown of circABCA1 (Fig. 2C). Subsequently, wound healing experiments indicated that ox-LDL enhanced the migration ability of VMSCs, but this effect was inhibited when si-circABCA1 was introduced (Fig. 2D). NF-κB pathway regulates inflammation and mediates the development of AS.^16^ This research, therefore, assessed inflammation by ELISA and immunoblotting. In fact, ox-LDL treatment induced increased levels of inflammatory factors and promoted the phosphorylation of NF-κB in VMSCs, while silencing circABCA1 reduced the inflammation in VMSCs (Fig. 2E‒F). During AS development, contractile VMSCs transform into secretory VMSCs and VMSCs acquire the ability to proliferate and migrate. Marker proteins for both phenotypes were subsequently assessed by immunoblotting. As measured, ox-LDL resulted in a decrease in α-SMA expression and an increase in OPN expression, but downregulating circVMSC rescued the changes in both proteins.

### CircABCA1 competitively binds miR-885–5p

**Fig. 3:**
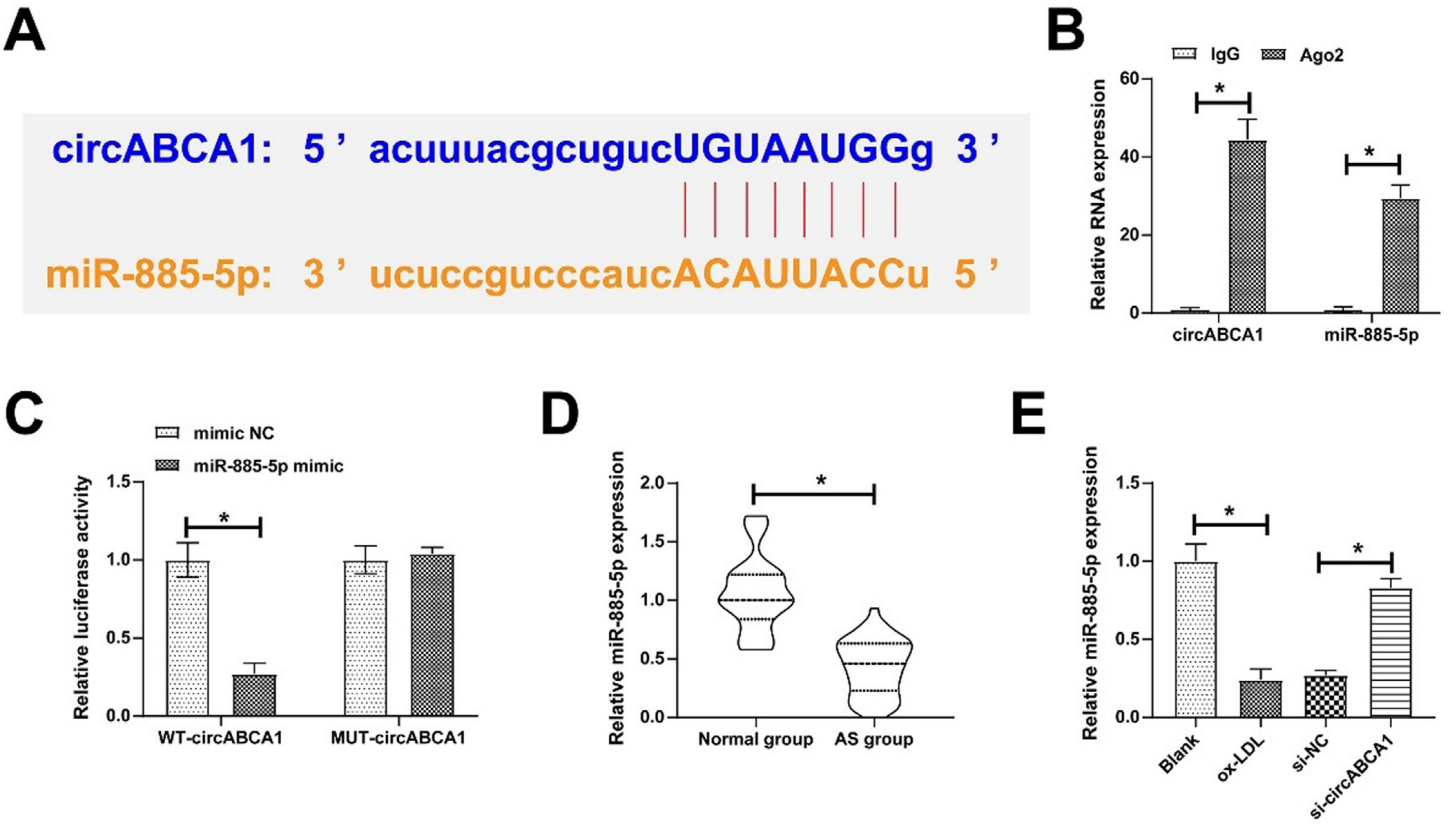
**CircABCA1 competitively binds to miR-885–5p.** Potential binding sites of circABCA1 and miR-885–5p on the https://circinteractome.nia.nih.gov/ (A), verification of circABCA1 and miR-885–5p targeting relationship (B‒C); miR-885–5p expression in patients’ sera (D) and VMSCs after knockdown of circABCA1 (E); data are expressed as mean ± SD (*n* = 3); *p < 0.05.

Through the bioinformatics website https://circinteractome.nia.nih.gov/, 10 miRNAs were found to share potential binding sites with circABCA1. RIP screening further targeted miR-885–5p as the study object because of its enrichment with circABCA1 in Ago2 magnetic beads (Fig. 3A‒B). Subsequently, the targeting relationship was further verified, as the results showed that WT-circABCA1 had a decreased luciferase activity when co-transfected with miR-885–5p mimic (Fig. 3C). Actually, miR-885–5p levels were lower in both AS patients and ox-LDL-treated VMSCs, but circABCA1 silencing restored miR-885–5p expression (Fig. 3D‒E).

### miR-885–5p blocks the promoting effects of circABCA1 on VMSCs

**Fig. 4:**
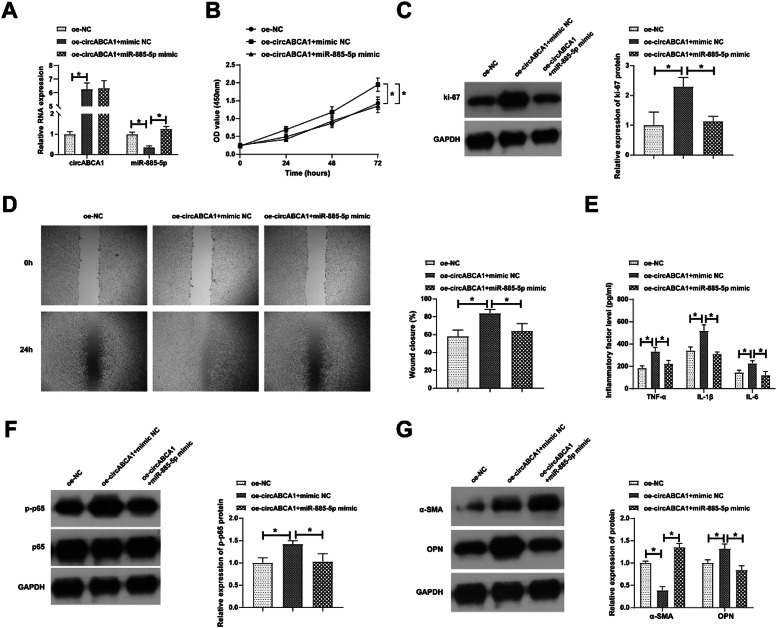
**miR-885–5p blocks the promoting effects of circABCA1 on VMSCs.** oe-circABCA1 and miR-885–5p mimic were co-transfected into ox-LDL-treated VMSCs. Changes in circABCA1 and miR-885–5p expression (A), proliferation (B), Ki-67 protein expression (C), migration ability (D), levels of inflammatory factors (E), p-NF-κB protein expression (F), α-SMA and OPN protein expression (G); data are expressed as mean ± SD (*n* = 3); *p < 0.05.

Based on a co-transfection design, ox-LDL-treated VMSCs were intervened with oe-circABCA1 and miR-885–5p mimic. As measured, oe-circABCA1 promoted circABCA1 and down-regulated miR-885–5p levels, while miR-885–5p mimic did not affect circABCA1 but restored miR-885–5p levels (Fig. 4A). Functional assays discovered the promoting effects of oe-circABCA1 on the proliferative, migratory, and inflammatory abilities of VMSCs, but concurrent induction of miR-885–5p reduced the changes in VMSC malignant proliferation, inflammation, and phenotypic switch (Fig. 4B‒G).

### ROCK2 is a target gene of miR-885–5p

**Fig. 5:**
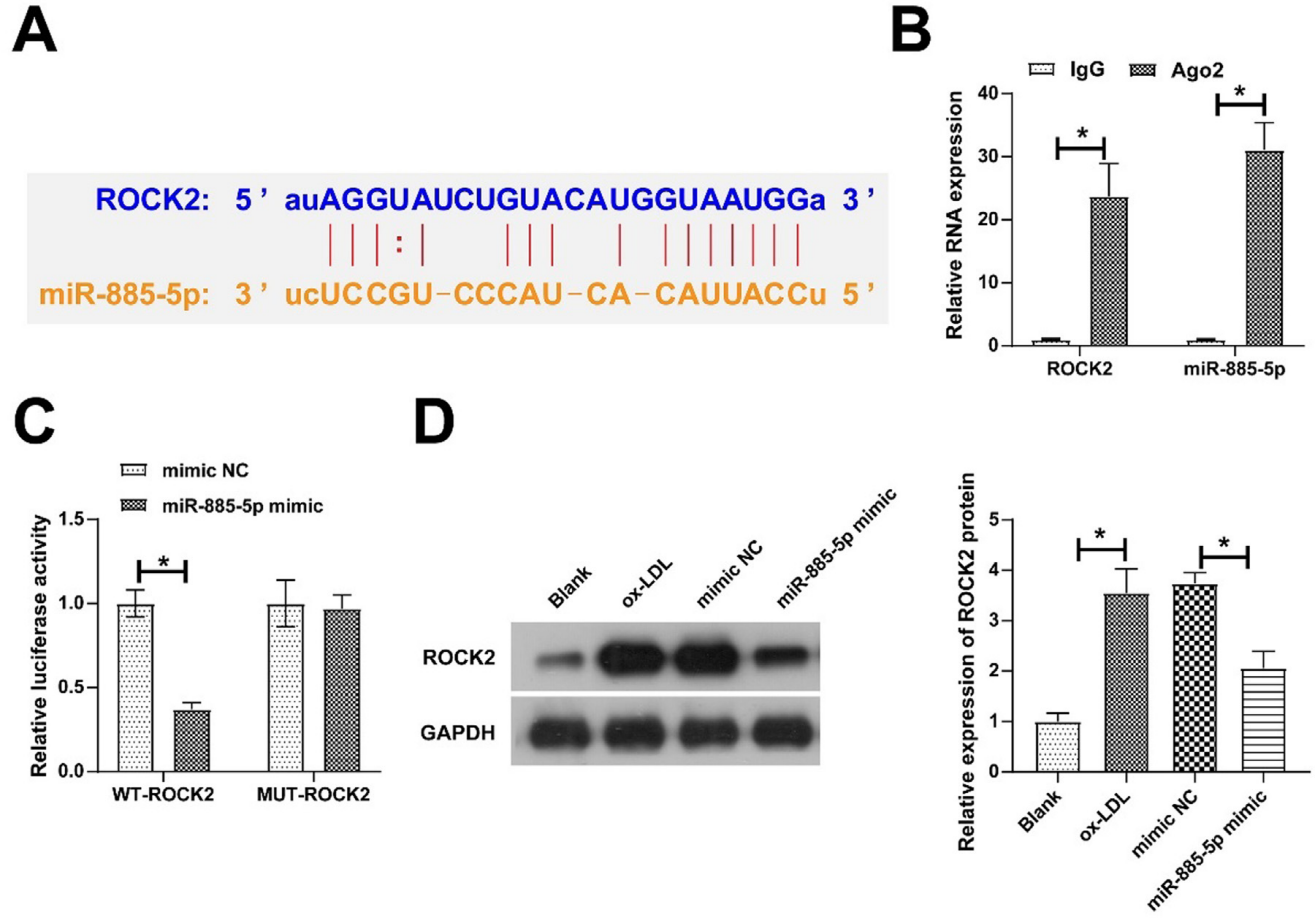
**ROCK2 is a target gene of miR-885–5p.** Potential binding sites of ROCK2 and miR-885–5p on the https://starbase.sysu.edu.cn (A), verification of ROCK2 and miR-885–5p targeting relationship (B-C); ROCK2 expression in VMSCs after overexpression of miR-885–5p (D); data are expressed as mean ± SD (*n* = 3); *p < 0.05.

Bioinformatics website found 10 target genes with potential binding sites to miR-885–5p, and ROCK2 and miR-885–5p were enriched in Ago2 magnetic beads by RIP screening (Fig. 5A‒B). The dual luciferase reporter assays further confirmed their targeting relationship as evidenced by miR-885–5p mimic-induced impairment of luciferase activity of WT-ROCK2 (Fig. 5C). Furthermore, oe-LDL induced ROCK2 expression, but this increase was prevented in the presence of miR-885–5p mimic in VMSCs (Fig. 5D).

### CircABCA1 regulates the miR-885–5p/ROCK2 axis in VMSCs

**Fig. 6:**
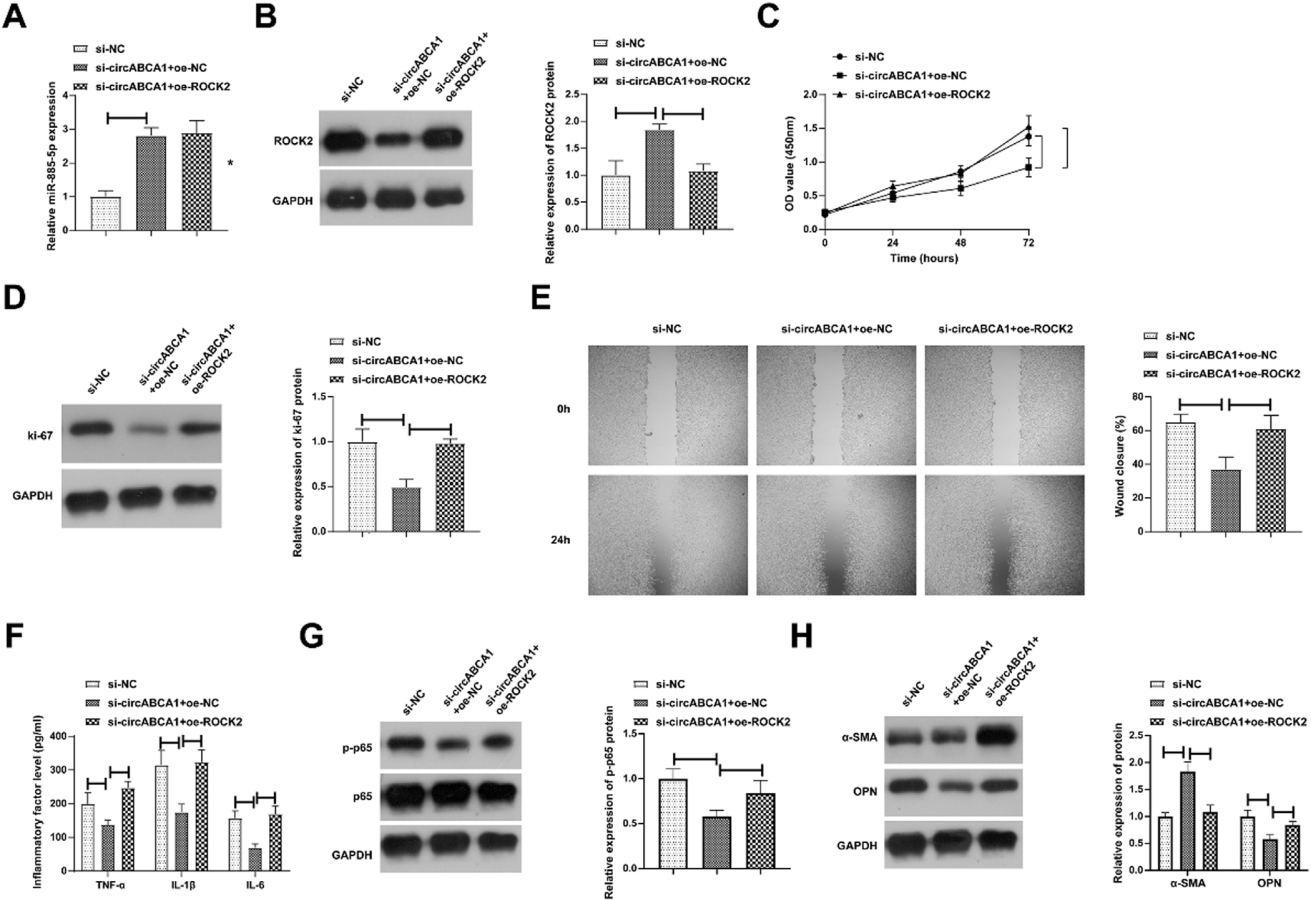
**CircABCA1 regulates the miR-885–5p/ROCK2 axis in VMSCs.** si-circABCA1 and oe-ROCK2 were co-transfected into ox-LDL-treated VMSCs. Changes in miR-885–5p and ROCK2 protein expression (A, B), proliferation (C), Ki-67 protein expression (D), migration ability (E), levels of inflammatory factors (F), p-NF-κB protein expression (G), α-SMA and OPN protein expression (H); data are expressed as mean ± SD (*n* = 3); *p < 0.05.

Subsequently, a co-transfection plan was implemented with si-circABCA1 and oe-ROCK2 into VMSCs. Fig. 6A and 6B checked the elevation of miR-885–5p and reduction of ROCK2 levels in VMSCs after si-circABCA1 introduction, while oe-ROCK2 did not affect miR-885–5p but restored ROCK2 levels. Functional experiments discovered that the effects of si-circABCA1 on VMSCs were reduced by oe-ROCK2, mainly manifested in the proliferation ability, Ki-67 protein expression, migration ability, inflammatory factor levels, NF-κB phosphorylation, and secretory transformation (Fig. 6C‒H).

### Knockdown of circABCA1 reduces arterial plaque size and improves inflammation in AS mice

**Fig. 7:**
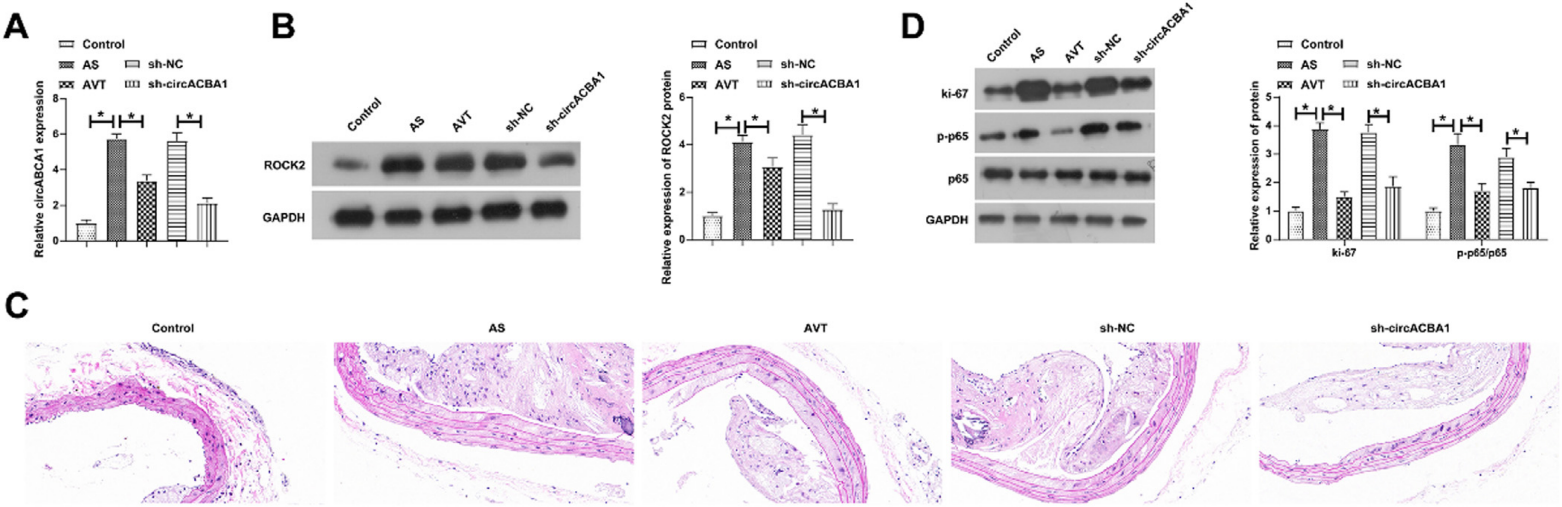
**Knockdown of circABCA1 reduces arterial plaque size and improves inflammation in AS mice.** circABCA1 and ROCK2 expression in aortic tissue (A‒B); HE staining to detect aortic pathological damage (C); IHC staining to assess the number of positive cells for OPN and α-SMA in the aorta (D); Ki-67 and P-NF-κB protein expression in the aorta (E); data are expressed as mean ± SD (*n* = 5); *p < 0.05.

To further support the *in vitro* results, *in vivo* experiments were next performed. AS mice were injected with sh-circABCA1, causing the decline of circABCA1 and ROCK2 levels in the aortic tissues. HE staining depicted that sh-circABCA1 reduced plaque size, and the treatment effect was close to that of atorvastatin treatment (Fig. 7C). IHC staining showed that sh-circABCA1 reduced the positive rate of OPN and promoted that of α-SMA in the aortic tissues (Fig. 7D). In addition, sh-circABCA1 also reduced Ki-67 and p-NF-κB expression in the aortic tissues (Fig. 7E).

## Discussion

Dysfunction of VSMCs has been accepted to be associated with the formation of AS.^12^ CircRNAs are a special class of regulators in various diseases including AS.^34^ Based on previous studies, this study treated VSMCs with ox-LDL to establish a cellular model of AS.^27^^,^^5^ The research discovered that circABCA1 was upregulated in AS patients, and moreover, circABCA1 was induced by ox-LDL in VSMCs in a time-dependent manner. Furthermore, the knockdown of circABCA1 suppressed AS progression *in vitro* and *in vivo* by suppressing VMSC malignant proliferation, inflammation, and phenotypic transformation through the miR-885–5p/ROCK2 axis.

CircRNAs are important regulators of VSMC proliferation, migration and inflammation, such as circ_GRN,^17^ circ_CHFR^39^ and circMAPK1.^7^ Abnormal proliferation of VSMCs can promote plaque formation in the early stage, but benefit in the late stage, such as preventing the rupture of the fibrous cap.^3^ VSMC phenotype switching has historically been considered a two-way process, with cells either adopting a physiological contractile phenotype or an alternative “synthetic” phenotype in response to injury.^36^ However, recent studies have believed that VSMC phenotype switching results in poorly differentiated forms lacking VSMC markers, including macrophage-like cells, responsible for the regulation of proliferation and chemotaxis-induced production of proinflammatory mediators, directly promoting AS.^4^^,^^1^ Therefore, modulating the phenotype of VSMCs in a direction favorable to alleviating disease progression is a promising therapeutic approach for AS. Here, circABCA1 was demonstrated as a novel regulator of VSMC phenotype switching and inflammation. The *in vitro* results showed that silencing circABCA1 inhibited the ability of VMSCs to proliferate and migrate and suppressed inflammatory response. Meanwhile, the present *in vivo* experimental results revealed that silencing circABCA1 inhibited the development of AS *in vivo*, similar to the therapeutic effect of atorvastatin.

Regarding the molecular mechanism of circRNAs in human pathogenesis, the most typical way for circRNAs is to absorb miR to regulate mRNA expression.^38^ The study indicated that miR-885–5p was a target of circABCA1. miR-885–5p is involved in various human diseases, especially different types of cancer,^26^ and is associated with chronic thromboembolic pulmonary hypertension^20^ and embryonic development.^22^ In the present study, miR-885–5p was downregulated in ox-LDL-induced VSMCs and was negatively regulated by circABCA1. Functional experiments showed that miR-885–5p prevented the promoting effects of overexpressed circABCA1 on VMSC proliferation, inflammation, and phenotypic transformation. To our knowledge, this is the first report that miR-885–5p mediates VMSC function in AS, based on the study by Streete L et al.^25^

To deeply understand the regulatory mechanism of miR-885–5p in AS, its target genes were predicted, and ROCK2 was identified. ROCK2 is a major regulator of central nervous system damage, and inhibition of ROCK2 can inhibit the activation of the NF-κB pathway and exert anti-apoptotic and anti-inflammatory roles.^18^ ROCK2 is also a key driver of cell migration^40^ and endothelial inflammation.^28^ Therefore, ROCK2 inhibition may be a potential therapeutic strategy for AS. The study measured that ROCK2 was up-regulated in ox-LDL-induced VSMCs, which was consistent with a previous study.^9^ Furthermore, ROCK2 suppressed circABCA1 up-regulation-induced protection against proliferation, migration, and inflammation of VSMCs.

Previous documents have elucidated that ROCK2 affects VSMC development by regulating multiple gene expression and signaling pathways, such as MLCP,^32^ JNK pathway,^30^ ERK, cyclin D1 and PCNA,^37^ whether circABCA1/miR-885–5p/ROCK2 axis regulates AS progression through these pathways requires further exploration. In addition, the mechanism by which ox-LDL induces circABCA1 upregulation in VSMCs is unclear.

## Conclusion

In conclusion, circABCA1 is stably expressed in VMSC as a circRNA, which upregulates ROCK2 expression by acting as a ceRNA for miR-885–5p. Knockdown of circABCA1 can inhibit malignant proliferation, inflammation and secretory phenotype transformation of VMSCs. However, circABCA1 mainly regulates miR-885–6p/ROCK2 axis to achieve this effect. These data provide strong data support for understanding the biological role of circRNA in the pathogenesis of AS and provide potential molecular targets for the treatment of AS.

## Authors’ contributions

Fang Yu and JiWei Liu Data curation, Fang Yu and Xiao Wei Formal analysis, JiWei Liu Investigation, Xiao Wei Methodology, Fang Yu Project administration, JiWei Liu Resources, Fang Yu Software, Xiao Wei Supervision, JiWei Liu Validation, Xiao Wei Visualization, Xiao Wei Writing - original draft, Fang Yu and JiWei Liu Writing, Fang Yu and JiWei Liu review & editing.

## Funding

Not applicable.

## Data available

Data is available from the corresponding author on request.

## Ethics approval

For human study, written informed consent and approval documents from the Ethics Committee of Yantaishan Hospital were obtained. Written informed consent was obtained from all patients. Animals underwent treatment with the approval of the Animal Ethics Committee of Yantaishan Hospital.

## Conflicts of Interest

The authors declare no conflicts of interest.
